# May the Number of Pregnancies Predict the Progression and the Outcome of Venous Disease Treatment?

**DOI:** 10.3390/diagnostics13152535

**Published:** 2023-07-30

**Authors:** Justyna Wilczko, Cezary Szary, Anna Bodziony, Krzysztof Celejewski, Siavash Swieczkowski-Feiz, Marcin Napierala, Dominika Plucinska, Jerzy Leszczynski, Michal Zawadzki, Tomasz Grzela

**Affiliations:** 1 Clinic of Phlebology, 02-034 Warsaw, Poland; 2 Center of Biostructure Research and the Department of General, Endocrinological and Vascular Surgery, Central University Hospital, Medical University of Warsaw, 02-091 Warsaw, Poland; 3 Maria Sklodowska-Curie National Research Institute of Oncology, 02-781 Warsaw, Poland; 4 Centre of Postgraduate Medical Education, 01-813 Warsaw, Poland

**Keywords:** embolization, pregnancy, lower limb venous insufficiency, pelvic venous insufficiency, venous disease, venous abnormalities

## Abstract

Pregnancy is a well-known risk factor for venous insufficiency. However, even nulliparous women experience venous problems. Therefore, we aimed to assess the possible associations between the number of pregnancies, veins condition and treatment outcome in women with venous disease. The retrospective assessment concerned data of 297 women with diagnosed venous insufficiency. Based on their pregnancy history, the patients’ records were divided into: nulliparous women (15.5%), those after 1–2 term pregnancies (57.9%) and those after ≥3 pregnancies (26.6%). The analysis concerned data from the diagnostics of the abdominal/pelvic and leg veins and the treatment results expressed as a symptoms/satisfaction score. Most of the nulliparous women developed venous disease due to mild anatomic abnormalities of the abdominal/pelvic veins. They responded to treatment (mostly unilateral embolization) very well. In the second group, the majority of the combined venous abnormalities responded to treatment with significant improvement, even after embolization alone, although nearly two-thirds still required further leg treatment. The third group was comprised of more advanced cases; nearly 40% of them with recurrence. In order to improve their condition, three-fourths of the cases required sequential treatment in both the pelvic and leg veins compartments. In conclusion, the number of pregnancies is a potent modifying factor in the pathogenesis of venous disease, especially in multiparous women. Together with abnormal venous anatomy, it may determine the treatment outcome.

## 1. Introduction

Pregnancy is recognized as an important risk factor for the development and progression of venous insufficiency [1]. In addition to hormonal changes, the most critical risk factors in pregnancy are significant hemodynamic abnormalities in the venous circulation, which occur in late pregnancy. The compression of abdominal vessels by the enlarged uterus results in blood stasis and the upstream dilatation of the pelvic veins. It further induces collateral circulation, occasionally visible as varicosities in the pudendal or groin regions and, subsequently, in the lower limb veins [2,3]. Although the majority of these changes are reversible, some of them persist and further enlarge, especially after each subsequent pregnancy. Hence, the failure of the pelvic venous system has been postulated as a presumable link between pregnancy and venous disease. However, it is well known that the occurrence of venous disease is not limited to multiparous women only, with it also affecting nulliparous women, albeit less frequently [1,4]. Although some explanations of this phenomenon have been proposed so far, the detailed mechanism remains unclear. Importantly, a better understanding of venous disease and its pathogenesis is crucial for more effective treatment, particularly considering the exceptionally high recurrence rate, as reported by some authors [5].

Therefore, the aim of our study was to analyze the possible association between the number of pregnancies, the condition of the venous system in the pelvic and lower limb compartments and the short-term outcome of treatment in women with venous disease.

## 2. Materials and Methods

The retrospective assessment involved previously collected data of patients with venous insufficiency, who underwent routine diagnostics and treatment in our clinic [6]. The study concerned females only to avoid sex-related data variability.

The medical database was searched using specified selection criteria: years range—2019–2021; sex—female; age—≥18; diagnosis—venous insufficiency with involvement of pelvic veins compartment; available reports from control visit, performed within 3 months of the last stage of venous treatment. The exclusion criteria concerned data of patients with thromboembolism, any neoplasm, advanced endometriosis, diagnosed inflammatory bowel disease or any other active inflammatory or unstable chronic disease that would disqualify the patient from endovascular procedures.

The extracted data set comprised all available records, including patients’ medical history, pre-operative reports of pelvic and lower limb venous system imaging, either from ultrasound scan and computed tomography- or magnetic resonance-venography, medical reports from all treatment procedures, as well as pre- and post-operative surveys with symptoms and patients’ satisfaction scores. The records extracted from the database were anonymized and subjected to further assessment.

All data were collected during routine procedures, according to the standard protocol of our clinic. Briefly, the sequential treatment was performed based on detailed pre-operative imaging of either the abdominal/pelvic or lower limb venous compartments. At the first stage, all patients who qualified for a treatment underwent transcutaneous endovascular pelvic veins embolization using Nester (Cook Medical, Bloomington, IN, USA) and/or Concerto (Medtronic/Micro Therapeutics Inc., Irvine, CA, USA) embolization coils under supervision of a C-arm fluoroscopy/X-ray device Zenition 70 (Philips Medical Systems, Best, The Netherlands).

**Figure 1:**
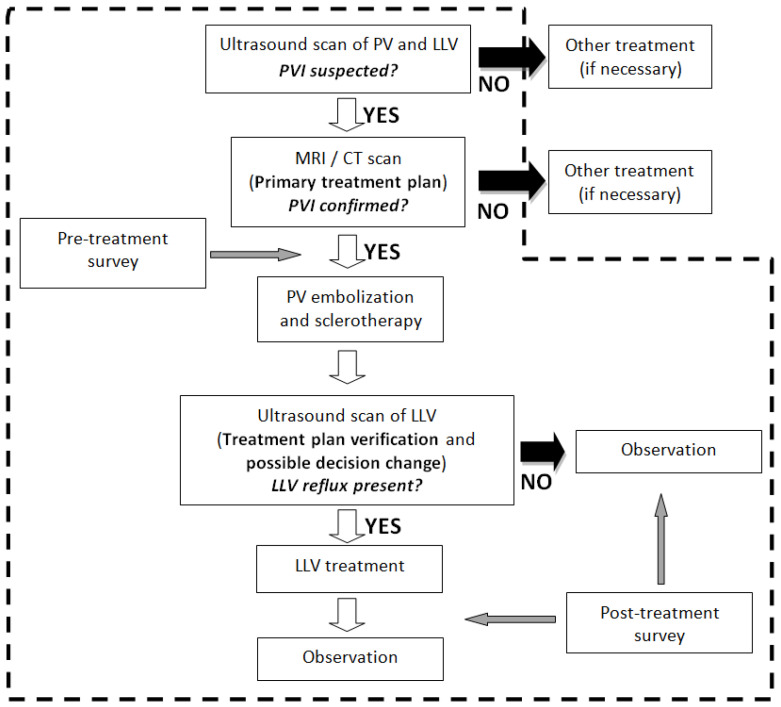
Diagnostics and treatment flow-chart (area marked with dashed borderline concerns the procedures included in analysis).

The supplementary ultrasound-guided foam sclerotherapy of pelvic “leakage” points was performed approximately 4–6 weeks after embolization. Then, depending on the primary qualification and result of the control ultrasound, patients were subjected to further treatment using laser thermoablation alone or in combination with phlebectomy or foam sclerotherapy [7,8]. Patients without clinically significant reflux (i.e., <0.5 s) in the lower limb veins were appointed for periodic control within 3 months of the last procedure. The flow chart with the diagnostics and treatment algorithm is shown on Figure 1.

The analysis also included reports from patients’ self-assessment of either pelvic/abdominal or lower limb pain/discomfort intensity, performed before and after treatment. Briefly, six main symptoms were evaluated using a 0–10 points visual analogue scale (VAS), each. Their sum was expressed as a total symptom score (TSS), with the minimum being 0 (no symptoms present) and maximum being 60 points (worst imaginable discomfort/pain). The TSS levels assessed before and after treatment were compared between groups. Furthermore, selected pelvic symptoms, including pelvic discomfort/pain at standing, sitting and at load/exercise were expressed as a mean of cumulated pelvic symptoms (MCPS) and compared among groups before and after treatment. The overall satisfaction from a treatment was assessed by each patient using the scale: “(1) fully satisfied”, “(2) satisfied”, “(3) unsatisfied without improvement” and “(4) unsatisfied with worsening”.

Although it is not required in retrospective studies, the concept of the study was submitted to the Local Ethics Committee (statement No. AKBE/181/2020).

The distribution of the analyzed variables was tested using the Shapiro-Wilk test with the rejecting null-hypothesis that the population is normally distributed at the *p*-value below the level 0.05. The baseline patients’ clinical characteristics, including age, number of deliveries and miscarriages, were analyzed using descriptive statistics with the calculation of the arithmetic mean, median and standard deviations, which were then compared between groups using an unpaired Student’s *t*-test. The variables concerning the occurrence of some features, including anatomic variants, previous treatment or distribution of patient satisfaction scores, were shown as respective ratios or percentages. Their statistical comparison between groups involved the Pearson’s chi-squared test. The possible associations between the selected parameters and the treatment outcome were evaluated using an odds ratio (OR), with 95% confidence interval (95% CI). The VAS scores, categorized as interval variables, were analyzed either individually or grouped as mean cumulated pelvic symptoms (MCPS) or a total symptoms score (TSS). They were assessed for each treated patient before and after the whole treatment using a paired Student’s *t*-test, as well as compared between groups using an unpaired Student’s *t*-test. The values of the patients’ satisfaction scores representing the ordinal scale were compared among groups using the Mann-Whitney U test. The observed differences were considered as statistically significant at *p* < 0.05. All calculations were performed using the MedCalc Software 18.11v (www.medcalc.org).

## 3. Results

The database search was performed according to the previously specified inclusion/exclusion criteria and resulted in the extraction of 297 records. Based on the patients’ previous pregnancy history, they were further divided into three groups: 46 nulliparous women (P0); 172 patients after one or two term pregnancies (P1-2) and 79 women after three or more (P3+) pregnancies. The selected baseline parameters, including the demography and some preoperative findings, differed among all of the groups and are briefly summarized in Table 1.

The P0 group (15.5% of the whole group) involved young women who were either nulliparous or those after early miscarriages (2.2% of the group). Almost two-thirds of them (60.9%) revealed some anatomic variants or abnormalities of varying clinical relevance concerning the left renal vein (LRV). Among others, they included various defects in the LRV outflow, with mild forms of the “nutcracker”-like phenomenon, retroaortic location of either the LRV or its branch draining blood from the left ovarian vein (LOV). Less frequent findings concerned abnormal drainage of the right ovarian vein (ROV) and some collateral circulation (Table 1). Before the treatment, a vast majority of the patients from this group reported relatively mild or moderate symptoms in their legs. The pain/discomfort in the abdominal/pelvic compartment and mean cumulated pelvic symptoms were moderate, although higher when compared to other groups (Table 2). In almost three-fourths of the patients from this group, the ovarian vein insufficiency was unilateral and concerned the left side only (I and II grade) [6]. More than one-third of the individuals presented hemodynamically and clinically significant LRV compression and required its additional balloon venoplasty. Two-thirds of the nulliparous women required the sequential treatment in both venous compartments, mainly those after previous unsuccessful interventions (21.7%). Importantly, although the odds of improvement in this group after sequential treatment were almost four-fold higher than after embolization alone, this predominance did not reach statistical significance, possibly due to the relatively small group size (OR = 3.9; 95% CI 0.90–16.8, *p* = 0.07). Moreover, it should be mentioned that although the mean TSS and mean pelvic symptoms intensity significantly decreased after treatment, they still remained significantly higher when compared to the other groups (Table 2). Nevertheless, 78.3% of the women from this group were satisfied with the treatment results, whereas 72.2% among them (56.6% of the group) assessed the improvement as significant (Table 2).

The P1-2 group (57.9% of all cases) comprised of significantly older women after one or two term pregnancies, with 7% of them having experienced early miscarriages in the past. The relatively mild venous abnormalities, although substantially similar to those found in the first group, differed in their frequency, especially in regard to the involvement of the ROV and/or internal iliac veins (IILV). Importantly, 30.2% of the cases from this group had already experienced previous interventions in the lower limb veins compartment. The ovarian veins’ dilatation and insufficiency were more pronounced (II and III grade) [6], mainly affecting the LOV (96.5% of cases). However, as mentioned previously, in 59.3% of the women from this group, the ROV was also involved (Table 1). Before the treatment, the patients reported moderate or mild abdominal/pelvic symptoms and moderate leg discomfort (Table 2). After the treatment, the mean scores of both the TSS and individual symptoms significantly decreased. The odds of achieving an improvement after the sequential treatment were almost two-and-half-fold higher compared to embolization alone (OR = 2.45; 95% CI 1.25–4.80, *p* = 0.01). Hence, 62.2% of the women from this group underwent the sequential treatment, especially those with recurrence. On the other hand, the remaining 37.8% of cases after pelvic veins embolization did not require auxiliary treatment, despite half of them preliminarily qualifying for that. Regardless of this group having the highest (29.7%) percentage of unsatisfied patients, including both subjects who declared worsening (0.7% of the whole group), 70.3% of the P1-2 women reported an improvement. Moreover, 73.6% of them (51.7% of the group) assessed this improvement as significant. Thus, although the individual satisfaction scores within this group were higher (i.e., worse) compared to the other groups, this difference was not significant (Table 2).

The third group (26.6% of all records) comprised of women after three or more (P3+) pregnancies. They were significantly older than the patients from the P0 group and similar to those from the second one (P1-2). More than one-third of them presented recurrent venous disease (38%), and this frequency was the highest among all of the groups. Similarly, the occurrence of early miscarriages in this group reached 41.2% and was significantly higher in comparison to the other groups. The ovarian vein insufficiency was bilateral (III-IV grade) [6] in three-fourths of the patients from this group, and this frequency was the highest among all of the groups (Table 1). Before the treatment, the women from this group reported mild abdominal/pelvic symptoms, with a significantly lower mean score compared to the nulliparous group, but not to the second group. The symptoms in the lower limb compartment were moderate and did not differ between the groups (Table 2). After embolization, 73.4% of the patients required a further auxiliary procedure. However, in 21.5% of the cases, the primary qualification was changed and leg treatment was, at least temporarily, abandoned. Hence, although the odds ratio of an improvement in this group favored the sequential treatment over the embolization alone (OR = 1.92; 95% CI 0.59–6.16, *p* = 0.27), this tendency did not reach statistical significance, possibly due to the relatively small group size. Importantly, despite the more advanced venous disease at baseline, including higher staging of pelvic veins insufficiency, as well as considerable alterations in the lower limb veins due to previous interventions, in the P3+ group, the improvement experienced by the patients after the treatment was most prominent. It included the lowest mean TSS and MPCS, as well as leg symptoms, and better satisfaction scores (although two latter did not reach statistical significance). After the entire treatment, 79.7% of the women from this group experienced the improvement, while 74.6% of them (59.5% of the group) assessed this improvement as significant (Table 2).

When the analysis was performed for the whole group, the improvement concerned 74.1% of the women; 73.6% of them reported it as significant. The odds ratio of an improvement in the whole group significantly favored the sequential treatment over the embolization alone (OR = 2.56; 95% CI 1.50–4.36, *p* = 0.0006). Noticeably, nearly one-third of all the assessed women had undergone some venous procedures in the past, and 80.4% of them, after pelvic veins embolization, still required some intervention in the lower limb veins. The estimated requirement for auxiliary procedures following embolization was significantly higher in patients with recurrence, compared to intact patients, with OR = 2.85, at 95% CI 1.58–5.12 and *p* = 0.0004.

## 4. Discussion

Previous studies have proven that the number of full term pregnancies/deliveries correlates with the risk of pelvic veins insufficiency (PVI) [1]. Therefore, women experiencing three or more term pregnancies/deliveries are much more prone to developing advanced venous disease and thus would require more complex treatment in both the pelvic and lower limb compartments [9]. Such a complex treatment might particularly be important in cases of previous unsuccessful interventions in the lower limb compartment, especially as the risk of recurrence is strongly associated with undiagnosed PVI [5]. Our data strongly suggest that the proper correction of PVI appears to be critical in this treatment. The beneficial role of pelvic veins embolization in sequential treatment may be supported by the observation that nearly one-fifth of the multiparous women experienced a significant improvement after embolization alone and did not require further intervention in the lower limb veins, even despite previous qualification for that. Regrettably, in cases of recurrence, due to alterations in the venous legs’ hemodynamics induced by previous interventions, the embolization alone may not suffice and further treatment remains necessary [9,10].

An interesting observation concerned the distribution of the pre-operative symptoms in the multiparous women [11,12]. They usually presented at least a moderate intensity of leg symptoms due to the more advanced venous disease in that compartment. On the other hand, the mean scores for the pelvic symptoms in this group were rather low and, especially when compared to nulliparous women, significantly lower despite relatively large morphological alterations in the abdominal/pelvic veins, including III and IV grade of PVI [6]. The presumable explanation for this peculiarity might be the fact that enlarged veins in the lower limb compartment could serve as a kind of compensation reservoir, which may attenuate the symptoms of pelvic veins overloading via pelvic “leakage” collaterals. Accordingly, the sequential treatment, when performed in both the pelvic and leg compartments, should provide the best outcome, especially in multiparous women. Indeed, in these patients, the mean values of the main post-operative scores, including the TSS, MCPS and general pelvic symptoms, as well as the satisfaction scores, were significantly better than in the other groups. Importantly, their mean post-operative score of leg symptoms was the lowest among the study groups, although this difference appeared to be non-significant.

There is no doubt in regard to the role of pregnancy in the pathogenesis of venous disease [1,13,14]. Nevertheless, even nulliparous women may develop some venous problems. However, as observed in our study, the origin of these problems was usually more complex and involved some abnormalities and/or anatomic variants of the venous system. All of these abnormalities were located in the abdominal/pelvic compartment, mainly affecting the left axis of the renal and ovarian veins. They included a broad spectrum of pathologies affecting the LRV outflow, with renal vein hypo- or dysplasia, intraluminal spurs, as well as extrinsic vein compression, better known as “nutcracker”-like phenomenon, retroaortic location of the LRV or abnormal drainage of the LOV. The majority of these morphological alterations, reported in the pre-operative imaging, were relatively mild, corresponding to the I or II grade of the PVI scoring scale [6]. However, they seemed disproportionate to the clinical manifestation, assessed using the symptoms scoring system. Importantly, the mean pre-operative intensity of pelvic symptoms in the P0 group was assessed as moderate, although it was the highest amongst the groups. After the treatment, the mean pelvic symptoms were reduced; nevertheless, they still remained higher than in other groups. Surprisingly, even though the overall advancement of venous disease in the lower limb veins of the nulliparous women was the lowest, and nearly one-third of the patients from that group did not require any intervention in that compartment, the mean preoperative leg symptoms were estimated as mild-to-moderate, and they did not differ from the other groups. Furthermore, although the mean score of leg symptoms significantly dropped following the treatment, it remained the highest among the study groups, but this difference appeared to be non-significant.

We have no easy explanation for the poor correlation between the clinical symptoms and preoperative image of the venous system observed in the P0 group. Also, the impression of the overall improvement was slightly aggravated by the higher mean symptoms intensity after the treatment. Presumably, the lower pain threshold in the nulliparous women than in the multiparous women should be considered [15,16]. Moreover, at least in several cases with more complex hemodynamic abnormalities, some residual overload of the pelvic veins could persist after incomplete correction [2,10,17]. However, some other possibilities cannot be excluded either.

While the nulliparous and multipregnant women represented two extreme states in regard to the condition of their venous system, the main features of both the P0 and P3+ groups overlapped in the women from the P1-2 group. As could be expected, in this group, the mean symptom scores, either before or after treatment, were also in-between, compared to the other groups. However, unexpectedly, nearly one-third of the women from this group were unsatisfied with the treatment results, including two patients who declared worsening. In addition to the patients’ too high expectations regarding the cosmetic outcome, the worst-to-treat cases concerned severe alterations in the venous anatomy and hemodynamics, especially after previous negligent venous surgery in the groin and/or upper thigh.

Regardless of the clear relationship between the number of pregnancies, the overall condition and the “treatability” of the venous system, our study also has some limitations.

The main limitation results from the retrospective nature of this study, which strongly restricted the pool of data available for analysis. The specific selection of patients could appear as the second issue. As our center specializes in the treatment of PVI, it should be emphasized that our cohort may not be considered as fully representative of the general population [18]. In addition, another limitation could be the short observation period. Obviously, to verify the longevity of improvement, a longer follow-up would be necessary, especially for patients for whom the decision regarding further LLV treatment was changed.

## 5. Conclusions

In our report, we describe an unsophisticated model for studies concerning the detrimental effect of pregnancy on the development and treatment of venous disease. This influence is especially prominent when occurring in women with mild anatomic abnormalities in the abdominal/pelvic veins. Thus, the rationale to include diagnostics and, if necessary, the correction of PVI as a key component of the hemodynamic treatment of venous disease seems to be of great importance, independent of the patients’ pregnancies history [18,19].

Our data confirm the previous observation that the number of pregnancies should be considered as a potent modifying factor in the pathogenesis of venous disease, especially in multiparous women [1]. Pregnancy can augment the primary impact of the abnormal venous anatomy in the pelvic compartment, although in multiparous women (after ≥3 pregnancies), it may be sufficient alone to promote venous disease development and progression. While each pregnancy reveals some detrimental effect on the pelvic and lower limb circulation, the number of full term pregnancies seems to correlate with the degree of damage to the veins in both compartments. Hence, the history of pregnancies may be useful to predict the outcome of treatment.

Based on the results of this study, we have formulated three “take-home messages”: (1) In nulliparous women, venous insufficiency in the lower limb compartment usually suggests abnormal anatomy (mainly LRV/LOV axis) in the abdominal/pelvic veins. The same applies to women who have experienced significant disease progression during or immediately after their first pregnancy. In addition to ovarian veins embolization, those patients may require LRV venoplasty and/or embolization of collaterals. (2) In multiparous (3+) women, LLV insufficiency usually results in more advanced PVI, although primary anatomic abnormalities in the abdominal/pelvic compartment are relatively rare. Patients from this group are more likely to benefit from embolization alone, even if they primarily qualified for both PV and LLV treatment. (3) Treatment after previous interventions in the LLV compartment is more challenging. However, multiparous women, including those with recurrent disease, may experience greater improvements in their symptoms compared to the other groups.

One has to keep in mind that our data originate from a group of specifically selected patients. Therefore, the aforementioned conclusions should be translated to the whole population with some caution. Further research—optimally a prospective multicenter study—should concern a larger group, focusing also on some potential confounders, including hormonal therapy, activity style, diet, etc. We believe such a study would fill the gap in the current understanding of the subject.

## Figures and Tables

**Table 1:** Clinical characteristics of study groups.

Feature/Parameter	Whole Group (*n* = 297)	P0 (*n* = 46)	P1-2 (*n* = 172)	P3+ (*n* = 79)
Age (Mean/median ± SD)	40.7/39.0 ± 9.1	34.2/33.0 ± 8.7	42.0/40.0 ± 8.9	41.6/40.0 ± 8.2
Deliveries (Mean/median ± SD)	1.7/2.0 ± 1.1	0.0/0.0	1.6/2.0 ± 0.5	2.9/3.0 ± 0.8
Miscarriages (n=; %)	46 (15.5%)	1 (2.2%)	12 (7.0%)	33 (41.8%)
Previous treatment (n=; %)	92 (30.9%)	10 (21.7%)	52 (30.2%)	30 (38.0%)
Main preoperative abnormalities (n=; %): LRV LOV ROV IILV Collateral circulation	68 (22.9%) 287 (96.6%) 171 (57.6%) 5 (1.7%) 13 (4.4%)	28 (60.9%) 45 (97.8%) 9 (19.6%) 0 (0%) 5 (10.9%)	34 (19.8%) 166 (96.5%) 102 (59.3%) 4 (2.3%) 6 (3.5%)	6 (7.6%) 76 (96.2%) 60 (75.9%) 1 (1.3%) 2 (2.5%)
Procedure planned/performed Pelvic veins only Pelvic AND leg veins	52/102 245/195	12/16 34/30	36/65 136/107	4/21 75/58
Decision change (n=; %)	50 (16.8%)	4 (8.7%)	29 (16.9%)	17 (21.5%)

Abbreviations: P0—nulliparous group, P1-2—women after 1–2 pregnancies, P3+ patients after 3 or more pregnancies, SD—standard deviation, LRV—left renal vein, LOV—left ovarian vein, ROV—right ovarian vein, IILV—internal iliac vein.

**Table 2:** Pre- and post-operative symptoms.

Feature/Parameter	Whole Group (n = 297)	P0 (n = 46)	P1-2 (n = 172)	P3+ (n = 79)
TSS (Mean/median ± SD): before treatment after treatment	24.3/24.0 ± 15.0 11.0/8.0 ± 9.6	25.7/27.0 ± 12.4 12.9/11.0 ± 8.5	24.5/24.0 ± 15.5 11.0/8.0 ± 10.0	23.0/23.0 ± 15.3 9.7/7.0 ± 9.2
Leg symptoms intensity (Mean/median ± SD): before treatment after treatment	4.9/5.0 ± 2.8 2.4/2.0 ± 2.2	5.0/5.0 ± 2.5 2.7/2.0 ± 2.3	5.0/5.0 ± 2.9 2.4/2.0 ± 2.2	4.6/5.0 ± 2.8 2.2/2.0 ± 2.1
General pelvic pain intensity: (Mean/median ± SD) before treatment after treatment	4.2/4.0 ± 3.4 1.8/1.0 ± 2.3	5.0/5.0 ± 3.2 2.5/2.0 ± 2.4	4.1/4.0 ± 3.4 1.8/1.0 ± 2.2	3.8/3.0 ± 3.5 1.5/0.0 ± 2.2
MCPS (Mean/median ± SD) before treatment after treatment	3.5/3.0 ± 3.2 1.4/0.8 ± 2.0	3.7/3.4 ± 3.1 1.8/1.3 ± 1.9	3.5/3.0 ± 3.2 1.5/0.8 ± 2.1	3.4/2.4 ± 3.2 1.1/0.3 ± 1.7
Satisfaction score (median of the group)	1.0	1.0	1.0	1.0
Satisfaction score distribution (n=; %) (1) fully satisfied (2) satisfied (3) not satisfied (no change) (4) not satisfied (worsening)	162 (54.5%) 58 (19.5%) 75 (25.3%) 2 (0.7%)	26 (56.6%) 10 (21.7%) 10 (21.7%) 0 (0%)	89 (51.7%) 32 (18.6%) 49 (28.5%) 2 (1.2%)	47 (59.5%) 16 (20.2%) 16 (20.2%) 0 (0%)

Abbreviations: P0—nulliparous group, P1-2—women after 1–2 pregnancies, P3+ patients after 3 or more pregnancies, SD—standard deviation, TSS—total symptoms score; MCPS—mean of cumulated pelvic symptoms.

## Data Availability

The original data are not publicly available due to restrictions aimed to protect patient confidentiality. The data presented in this paper are available from the corresponding author upon request.
